# Mitochondrial Control and Guidance of Cellular Activities of T Cells

**DOI:** 10.3389/fimmu.2017.00473

**Published:** 2017-04-24

**Authors:** Tung Chao, Haiping Wang, Ping-Chih Ho

**Affiliations:** ^1^Department of Fundamental Oncology, University of Lausanne, Lausanne, Switzerland; ^2^Ludwig Center for Cancer Research, University of Lausanne, Lausanne, Switzerland

**Keywords:** immunometabolism, mitochondrion, macrophage, T cell, oxidative metabolism

## Abstract

Immune cells protect us against infection and cancer cells, as well as functioning during healing processes to support tissue repairing and regeneration. These behaviors require that upon stimulation from immune activation the appropriate subsets of immune cells are generated. In addition to activation-induced signaling cascades, metabolic reprogramming (profound changes in metabolic pathways) also provides a novel form of regulation to control the formation of desirable immune responses. Immune cells encounter various nutrient compositions by circulating in bloodstream and infiltrating into peripheral tissues; therefore, proper engagement of metabolic pathways is critical to fulfill the metabolic demands of immune cells. Metabolic pathways are tightly regulated mainly *via* mitochondrial dynamics and the activities of the tricarboxylic acid cycle and the electron transport chain. In this review, we will discuss how metabolic reprogramming influences activation, effector functions, and lineage polarization in T cells, with a particular focus on mitochondria-regulated metabolic checkpoints. Additionally, we will further explore how in various diseases deregulation and manipulation of mitochondrial regulation can occur and be exploited. Furthermore, we will discuss how this knowledge can facilitate the design of immunotherapies.

## Introduction

Immune cells circulate throughout our bodies and reside in peripheral tissues where they perform many vital functions, including providing protection against pathogens, impeding cancer cells, and supporting tissue homeostasis. How immune cells respond to the nutrient state of various tissues and whether their nutrient-sensing mechanisms regulate immune responses are fascinating questions yet to be resolved. Research accumulated in the past decade has revealed that immune cells change their metabolic programs to support activation and differentiation (1, 2). Moreover, protective immune responses may be detrimentally affected by metabolic stress caused by nutrient deprivation, coinhibitory receptors, and production of certain metabolites. By contrast, targeting the metabolic machineries that immune cells exploit during activation can lead to promising outcomes with improved antitumor immunity and autoimmunity in preclinical animal models (3, 4). These findings expose the importance of metabolic reprogramming in guiding immune responses and suggest that the nutrient composition of where the immune cell resides and infiltrates may be a critical regulator in orchestrating immune responses.

Mitochondrion is the metabolic hub of the cell that governs energy production through coordination of the electron transport chain (ETC) and the tricarboxylic acid (TCA) cycle. In addition to energy production, mitochondrion catabolizes nutrients, including glucose, amino acids, and fatty acids, to produce building blocks for cell growth and expansion (5). In order to meet their metabolic demands, cells have to change their mitochondrial dynamics, including mitochondrial volume, structure, membrane potential (ΔΨm), and location, in response to nutrient availability and growth stimuli. Although research on how mitochondrial dynamics impact cellular metabolism have been mainly conducted in cancer and stem cells (6–8), several recent studies have revealed that this process may play critical roles in immunometabolic regulation in both innate and adaptive immune cells (9). In this review, we will summarize how T cells control mitochondrial dynamics in order to meet their metabolic demands. Furthermore, we will discuss how mitochondria-derived signals potentially guide T cell activation and differentiation.

## Mitochondrion: The Central Regulator of Cellular Metabolism and Beyond

### Mitochondria Are the Power Plant for Cellular Bioenergetic Demands

Mitochondria are the major energy producers within a cell that couple metabolite oxidation to aerobic respiration (10). Glucose and fatty acids, after being catabolized through glycolysis and β-oxidation, respectively, fuel the TCA cycle in the form of acetyl-CoA. Acetyl-CoA is further oxidized into carbon dioxide to generate NADH and FADH_2_, the main sources of electrons for the ETC. The ETC ultimately transfers electrons provided by NADH and FADH_2_ to oxygen, while concurrently generating ΔΨm across the mitochondrial inner membrane. This proton gradient is further utilized to produce ATP (9). In addition to generating ATP, mitochondria also contribute to lipid and amino acid synthesis to build macromolecules. Acetyl-CoA can be transported out of the mitochondria into the cytosol where it is used for protein acetylation as well as *de novo* fatty acid synthesis (11). Citrate can also be exported into the cytosol for *de novo* fatty acid synthesis and into the nucleus to serve as an acetyl-CoA donor for histone modification (12). Furthermore, the metabolic intermediates of the TCA cycle can also be used for production of cholesterol, nucleotides, and amino acids. These processes provide building blocks for cell proliferation and growth at a cost of TCA cycle metabolite depletion. Altogether, mitochondria bridge nutrient metabolism to fulfill the bioenergetic demands of cell through the coordination of TCA cycle and ETC.

### Mitochondrial Dynamics under Metabolic Stress and Reprogramming

Mitochondrial quality and activities are maintained and adjusted through mitochondrial dynamics. In response to the type, strength, and duration of metabolic or genomic stress, mitochondrial dynamics regulate mitochondrial fusion, fission, mobility, biogenesis, and degradation. Mitochondrial mobility regulates the subcellular localization of mitochondria, whereas mitochondrial fusion and fission controls mitochondria mass and metabolism by fusing (fusion) or splitting (fission) the inner and outer membranes and matrix components (13, 14). Nutrient deprivation induces the formation of a tubular network of mitochondria by promoting mitochondrial fusion and suppressing mitophagy (a mitochondrial clearance process) (15). By contrast, severe and prolonged DNA damage leads to mitochondrial fission and further facilitates the clearance of damaged mitochondria *via* mitophagy (16). Thus, mitochondrial fusion and fission provide a method to efficiently and flexibly adjust mitochondrial quality and quantity. Importantly, most of the mitochondrial fusion and fission machineries are conserved from yeast to mammals, further implicating the importance of these processes (17). Mechanistically, mitofusin and optic atrophy 1 (OPA1) are two dynamin-like GTPases that control fusion of the mitochondrial outer and inner membranes, respectively (18). These proteins are regulated by ubiquitination and proteolytic cleavage. When mitophagy is induced, mitofusin 1 and 2 are ubiquitinated in a PTEN-induced putative kinase 1/Parkin-mediated manner (19). Moreover, OPA1 is constitutively cleaved by protease Yme1L in the intermembrane space in order to shape proper cristae structures (20). Furthermore, loss of mitochondrial ΔΨm induces OPA1 cleavage by protease OMA1, a process that further dampens mitochondrial fusion (21, 22). Conversely, mitochondrial fission is triggered by phosphorylation of dynamin-related protein 1 (Drp1) on serine 616 by protein kinase C. This event promotes Drp1 translocation to the mitochondrial outer membrane and facilitates the association between Drp1 with other adaptor proteins, including Fis1 (mitochondrial fission 1 protein), Mff (mitochondrial fission factor), and MiD49/51 (23–25). By contrast, phosphorylation of Drp1 on serine 637 by protein kinase A (PKA) leads to Drp1 inactivation (26). Additionally, mitofusin 2 is regulated *via* JNK phosphorylation, which when coordinated with Huwe1-regulated ubiquitination promotes stress-induced mitochondrial fragmentation and apoptotic cell death (27). These regulatory steps tightly control the balance of mitochondrial fusion and fission to actively fine-tune the mitochondria’s metabolic activity. Mitochondrial fusion can increase cristae formation and respiratory complex formation as well as increasing the substrate uptake fueling oxidative phosphorylation (OXPHOS). Moreover, fusion also promotes fatty acid oxidation (FAO), which is important for the formation and survival of memory T cells (discussed in Section “Metabolic Reprogramming Fuels T Cell Activation and Differentiation”) (28). On the other hand, mitochondrial fission not only acts to eliminate dysfunctional mitochondria but is also an adaptation that occurs in response to increased aerobic glycolysis (29).

## Metabolic Reprogramming Fuels T Cell Activation and Differentiation

During viral infection, T cell activation occurs *via* several distinct phases. Cell growth is the initial phase, with subsequent massive clonal expansion and differentiation, followed by an abrupt contraction phase, and then persistent long-lived memory T cells (30). During the initial growth phase, T cells undergo an activation-induced reprogramming of their metabolism. FAO in naive T cells is converted to anabolic metabolism in activated T cells, including increased aerobic glycolysis, pentose-phosphate pathway, and glutaminolysis (31, 32). Mitochondrial biogenesis and proteomic reprogramming are induced upon T cell receptor (TCR) activation in T cells. Mitochondrial regulations strongly activate one-carbon metabolism fueled by serine and glycine metabolism, which is essential for redox homeostasis as well as purine and thymidine formation (33, 34). In contrast to cytotoxic effector T cells, memory T cells possess elevated mitochondrial mass and use fatty acids as the main energy source through FAO (35, 36). Interestingly, helper effector T cells (Th1, Th2, or Th17) use aerobic glycolysis to support their effector functions and differentiation programs similar to cytotoxic CD8^+^ T cells, whereas regulatory T cells utilize FAO to maintain lineage stability and immunosuppressive functions (37–39). Several recent reviews have summarized how these metabolic pathways intertwine with T cell activation and differentiation (3, 40). Therefore, we will focus on how mitochondria-derived regulations are involved in these processes.

### Mitochondria-Governed Metabolic Regulations in Memory T Cells

**Figure 1:**
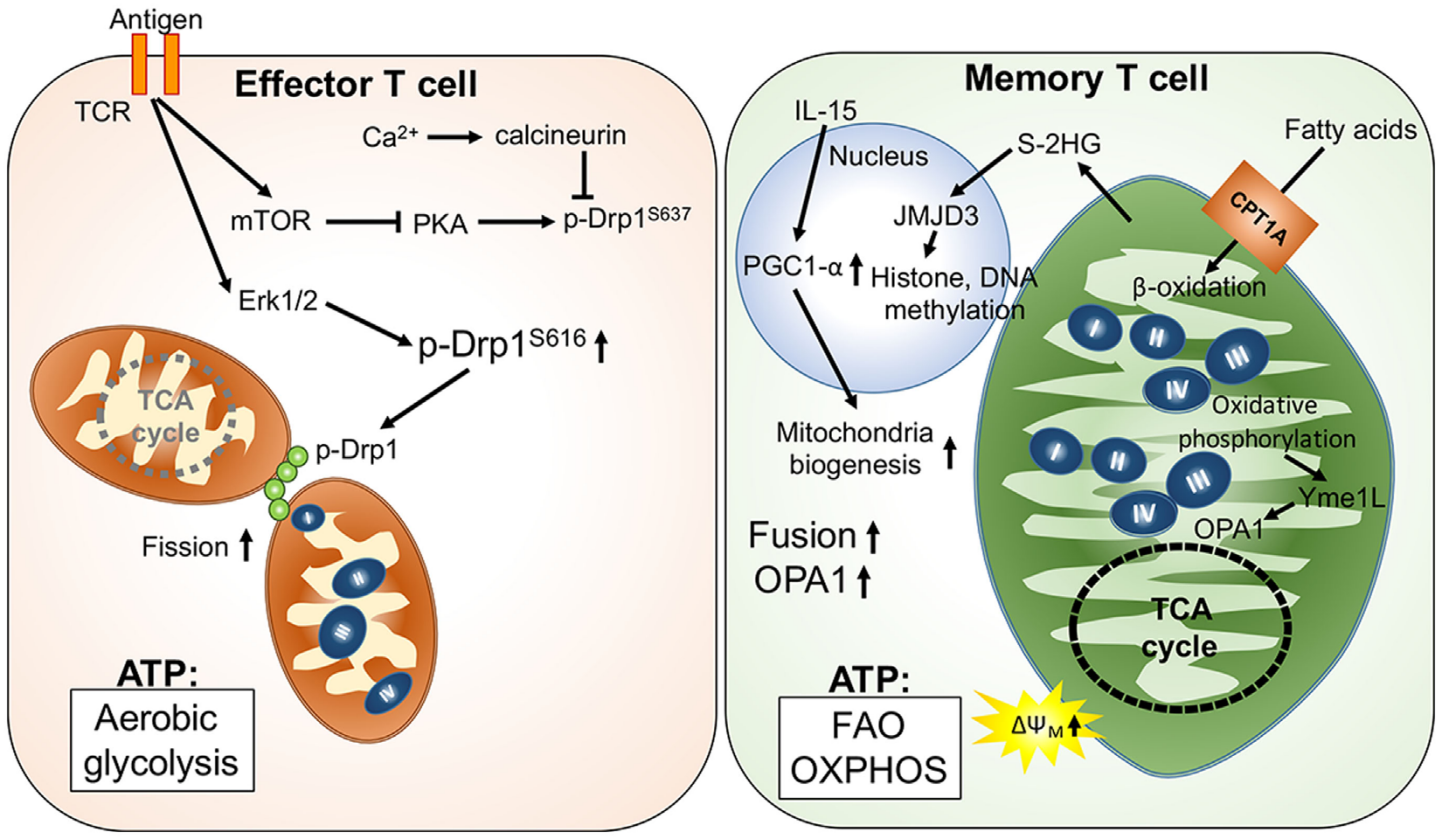
**Mitochondria-governed metabolic regulations upon T cell activation and memory T cell formation**. T cell receptor (TCR) activation signaling facilitates mitochondrial fission, leading to looser cristae structures, and lower respiratory functions. In this scenario, ATP production is mainly from aerobic glycolysis. By contrast, fusion of mitochondria is facilitated by upregulation of optic atrophy 1 (OPA1)-mediated mitochondrial inner membrane fusion in memory T cells. Furthermore, the tight cristae structure and compact respiratory complex also support maintenance of higher mitochondrial membrane potential (ΔΨm) and the oxidative metabolism in mitochondria.

Unlike the short-lived effector T cells that rely on aerobic glycolysis, memory T cells display increased mitochondrial mass, spare respiratory capacity, and utilize FAO to meet their metabolic demands (35, 41). Reduced glucose uptake and glycolysis has been shown to not only suppress effector T cell functions but also promote T cell differentiation toward memory-like precursor cells that have an elevated dependence on mitochondrial metabolism (42). Additionally, it has been shown that IL-15, an important cytokine for the survival of memory T cells, increases mitochondrial mass and copy numbers in memory T cells (35, 43). Furthermore, the elevated oxidative metabolism and mitochondrial activity in memory T cells could positively impact survival and the rapid transition to glycolysis after re-stimulation (36, 41, 44). These findings suggest that mitochondrial mass and activity play critical roles in guiding the generation of memory T cells. Interestingly, mitochondrial dynamics have also been reported to modulate the differentiation process in effector and memory T cells. Effector T cells primarily possess fragmented mitochondria, which might facilitate aerobic glycolysis. During TCR activation by antigen-presenting cells, Drp1-mediated mitochondrial fission and translocation of mitochondria to the immune synapse could be promoted by suppression of PKA-induced Drp1 phosphorylation at serine 637 by mTOR activation and the calcium (Ca^2+^) flux-mediated phosphatase calcineurin (45–48). By contrast, Opa1-dependent mitochondrial fusion promotes memory T cell formation and oxidative metabolism by maintaining the tightly associated cristae structures and ETC complexes (Figure 1). Notably, OXPHOS can stimulate mitochondrial inner membrane fusion through Yme1L-mediated cleavage of Opa1 (49). In addition, oxidative stress-induced glutathione oxidation results in elevated mitochondrial fusion by promoting mitofusin dimerization (50). Moreover, mitochondrial deacetylase sirtuin-3 (SIRT3) deacetylates OPA1, which activates OPA1’s GTPase activity promoting mitochondrial networking and preventing cell death (51). Thus, TCR signaling and metabolites derived from oxidative metabolism contribute to the regulation of T cell mitochondrial dynamics.

In addition to regulating mitochondrial structure, metabolites derived from mitochondrial metabolic pathways may also regulate T cell differentiation by affecting the epigenetic and transcriptomic landscape. One recent study demonstrated that S-2-hydroxyglutarate (S-2HG) can act as an immunometabolite altering the gene expression profile with changes in the epigenetic landscape toward memory-like CD8^+^ T cells (52). Since S-2HG is a chemical analog of α-ketoglutarate and succinate, we postulate that production of α-ketoglutarate and succinate could regulate T cell differentiation by modulating Jumonji D3 (Jmjd3)- and ten eleven translocation (Tet)-dependent histone and DNA methylation, respectively (53, 54). These mitochondria-derived metabolites may provide signals that impact differentiation, exhaustion, and the secretome of T cells through mitochondria–nucleus communication. Thus, future investigations to determine if mitochondrial dynamics and activity can modulate T cell differentiation through as yet undefined mitochondria–nucleus communication will provide exciting new insights for immunometabolic regulation.

### T Cell Exhaustion during Viral Infection and in the Tumor Microenvironment

T cell exhaustion describes a state when T cells are incapable of proliferating or producing effector molecules, which often occurs as a result of chronic antigen exposure in diseases such as viral infections and tumors (55). Although changes in the transcription program of T cells can contribute to T cell exhaustion, recent studies have revealed that metabolic insufficiency and deregulation of nutrient-sensing pathways also contribute to T cell exhaustion (56). During chronic lymphocytic choriomeningitis virus infection, Bengsch et al. reported that glycolytic and mitochondrial metabolism in early effector CD8^+^ T cells are repressed by programmed cell death protein 1 (PD-1) signaling. PD-1 signals also suppress the expression of peroxisome proliferator-activated receptor gamma coactivator 1-alpha (PGC1α) that inhibits mitochondrial biogenesis in viral-specific CD8^+^ T cells leading to declined effector function in exhausted T cells (57). Intriguingly, PGC1α overexpression in exhausted T cells is able to enhance their effector functions and improve mitochondrial biogenesis. By contrast, it has been reported that HIV-specific CD8^+^ T cells display increased mitochondrial mass, resulting in higher cluster of differentiation 95 (CD95)/CD95-ligand induced apoptosis (28, 58, 59). Thus, it is important to further investigate the contributions of mitochondrial biogenesis on T cell exhaustion and how we can target mitochondrial metabolism of T cells when treating chronic viral infection.

**Figure 2:**
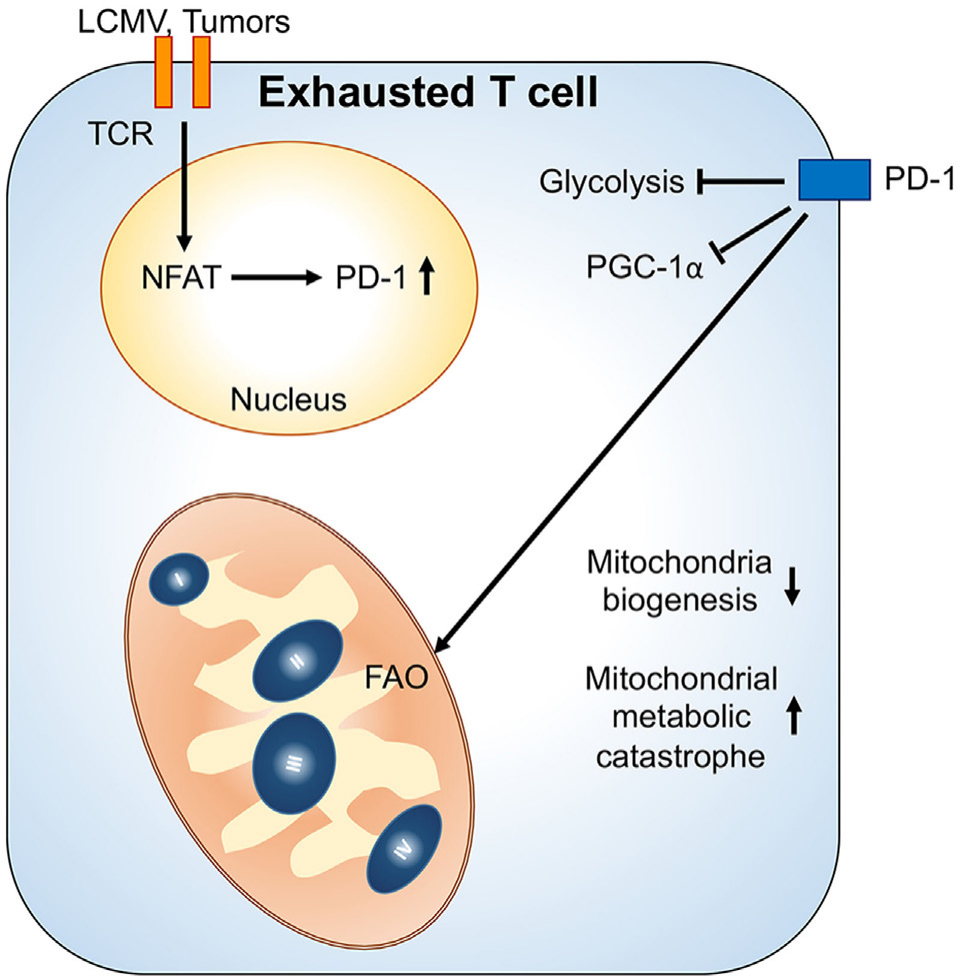
**Mitochondrial metabolic reprogramming under exhaustion of T cells**. Upon chronic antigenic stresses such as lymphocytic choriomeningitis virus (LCMV) clone 13 infection and tumors, sustained T cell receptor (TCR) activation leads to NFAT-mediated programmed cell death protein 1 (PD-1) upregulation. Upregulation of PD-1 and chronic TCR activation suppress PGC1α expression, leading to decrease of mitochondrial biogenesis. Moreover, PD-1 signal would suppress glycolysis and promote mitochondrial metabolism toward fatty acid oxidation (FAO). These metabolic reprogramming in mitochondria may further cause mitochondrial metabolic catastrophe if there is overload of mitochondrial oxidative metabolisms without sufficient antioxidant responses.

The tumor microenvironment can also cause metabolic insufficiency in T cells by reducing PGC1α-mediated mitochondrial biogenesis (60). Similar to chronic viral infection, overexpressing PGC1α in tumor antigen-specific CD8^+^ T cells sustains their metabolic fitness and improves their antitumor responses in the tumor microenvironment. Of note, PD-1 signal is dispensable in tumor-induced impairment of mitochondrial biogenesis in T cells. Since hypoxia and nutrient deprivation caused by elevated tumor metabolism could drastically affect mitochondrial metabolism and dynamics (61), it is likely that T cells fail to sustain their mitochondrial activity and biogenesis due to a metabolic status in the tumor microenvironment that does not exist in the chronic viral infection condition. Interestingly, PD-1 signaling has been shown to increase FAO and mitochondrial spare respiratory capacity in effector T cells (62). Therefore, we postulate that the impairment in mitochondrial biogenesis while receiving PD-1 signaling might result in cell death and senescence in tumor-infiltrating T cell due to metabolic catastrophe (Figure 2). Altogether, these recent studies in chronic viral infection and tumors suggest that mitochondrial metabolism in T cells could critically regulate T cell immune responses in disease progression. Further investigations are needed to reveal how mitochondrial activity is connected with T cell immune responses and whether regulation of mitochondrial dynamics also controls T cells behavior. These investigations will provide novel insights for immunometabolic regulation and facilitate the design of immunotherapies.

### T Cell Aging

Accumulation of dysfunctional mitochondria is frequently observed in aged cells (63). The mitochondrial free radical theory of aging has been recognized for decades (64); oxidation of cellular macromolecules and mitochondrial DNA damages are thought to be mainly caused by reactive oxygen species (ROS) produced from dysfunctional mitochondria (65, 66). Aged T cells display a reduction in respiratory metabolism and ETC activity; this is accompanied by reduced Ca^2+^ influx into mitochondria and elevated mitochondrial ROS production. These changes could lead to decreased ΔΨm and ATP production, as well as activation of nuclear factor of activated T-cells (67). Aged T cells may also upregulate the expression levels of coinhibitory receptors such as PD-1, TIGIT (T-cell immunoreceptor with Ig and ITIM domains), Lag-3 (lymphocyte-activation protein 3), and Tim-3 (T-cell immunoglobulin and mucin-domain containing-3). Therefore, two attractive strategies to rejuvenate the immune responses of aged T cells are (1) activate mitochondrial biogenesis and (2) improve mitophagy-mediated mitochondrial quality control. Among the proposed strategies, nicotinamide adenosine dinucleotide (NAD)-dependent regulation has delivered promising outcomes in different aging studies (68). Manipulating cellular metabolism or treating with nicotinamide riboside increases NAD and may prevent aging by restoring mitochondrial activity. The restoration of mitochondrial activity occurs mainly because of mitochondrial biogenesis activation induced by a sirtuin 1–PGC1α pathway and SIRT3-induced mitophagy (69, 70). However, it remains unclear if these strategies can also be applied to aged T cells. Detailed analysis of the contributions of deregulated mitochondrial dynamics in T cell aging will provide a springboard for developing immunometabolic boosters to rejuvenate aged T cells.

### T Cells in Autoimmunity

A well-functioning immune system is reliant on the selection of a lymphocyte repertoire that is adequately diverse in response to numerous foreign antigens yet sufficiently self-tolerant to avoid the development of autoimmunity. As described above, production of ROS and ATP synthesis, which are tightly associated with the ΔΨm, regulate the activation, proliferation, and selection of the cell death pathway in T cells. Recent evidence has proposed that the regulation of programmed cell death in T cells is impaired in both human and murine systemic lupus erythematosus (SLE) contributing to other disease pathogenesis (71). Meanwhile, ΔΨm and ROS levels are elevated in patients with SLE in comparison with healthy controls (72). When mitochondrial ROS was blocked in lupus T cells, mice showed reduced interferon pathway signaling and had decreased signs of autoimmune disease (73). Furthermore, coordinated mitochondrial hyperpolarization and ATP depletion play key roles in abnormal T cell death in lupus patients (74). Hence, persistent mitochondrial hyperpolarization, which leads to increased ROS production and ATP depletion, might be responsible for the unusual increase in spontaneous apoptosis in SLE patients and other relative autoimmune diseases. However, the relative impact and metabolic signaling pathways involved still require further investigation. This will likely allow us to identify novel targets for pharmacological intervention in patients with autoimmune diseases.

## Conclusion Remarks

Over the past two decades, studies have revealed that metabolic checkpoints couple metabolic demands and activation signaling cascades to orchestrate immune cell differentiation, proliferation, survival, and function, through multiple layers of regulation. These regulations are operated through the most ancient cellular machineries to ensure proper immune responses are engaged. In this review, we discuss how mitochondrial dynamics can support T cell metabolic demands and the contribution of mitochondrial dynamics to T cell behavior in different diseases. Nevertheless, the detailed regulation and contribution of mitochondrial dynamics to immune cells remains to be fully explored. Most importantly, deciphering how mitochondrial activity communicates with the nuclear landscape, including changes in epigenetics and the transcriptome, will provide novel information regarding immunometabolic regulation and cell biology. We also envisage that these mitochondria-derived regulations may be utilized in other immune cells, including antigen-presenting cells, NK cells, and innate lymphocytes. Altogether, understanding these underlying immunometabolic controls will provide the foundations for novel immunotherapies that can selectively tune the immune responses of deregulated immune cells in various diseases, including autoimmunity and cancer.

## Author Contributions

All authors listed have made substantial, direct, and intellectual contribution to the work and approved it for publication.

## Conflict of Interest Statement

The authors declare no potential conflict of interest with any commercial or financial relationship on this work. The reviewer, MG, and handling editor declared their shared affiliation, and the handling editor states that the process nevertheless met the standards of a fair and objective review.
